# Age matters:  Young T lymphocytes offer better protection from myeloma proliferation

**DOI:** 10.1186/1742-4933-10-5

**Published:** 2013-02-18

**Authors:** Alexander F Glick, Yan S Song, Brian Hwang, John Lillvis, Pat Zanzonico, Camil Fuchs, Roger N Pearse, Paul Szabo, Marc E Weksler

**Affiliations:** 1 Department of Medicine, Weill Cornell Medical College, New York, NY, USA; 2 Department of Medical Physics, Memorial Sloan-Kettering Cancer Center, New York, NY, USA; 3 Department of Statistics, Tel Aviv University, Tel Aviv, Israel; 4 Department of Neurology, Weill Cornell Medical College, New York, NY, USA

## Abstract

**Background:**

The incidence and growth of cancer has been reported to increase with age and/or impaired T lymphocyte function.

**Results:**

Consistent with these observations, we found that a monoclonal serum immunoglobulin (mIgG2b), rarely detectable after the injection of 5T33 murine multiple myeloma (MMM) cells into 3–4 month old wild-type C57BL/6 mice was seen more frequently in 18–20 month old wild-type C57BL/6 mice and in 3–4 month old Rag1-deficient C57BL/6 mice. These observations were confirmed and extended using more sensitive assays such as quantitation of splenic mRNA specific for the canonical 5T33 monoclonal IgG2b produced by 5T33 myeloma cells and the most sensitive assay, photon-imaging of mice injected with 5T33 cells, stably transfected with fire-fly luciferase gene (5T33L cells), which emit photons after the injection of luciferin. Furthermore, the proliferation of 5T33L myeloma cells in Rag1-deficient C57BL/6 mice was greater in mice which also received spleen T cells from 18–20 month old C57BL/6 wild-type mice compared to mice which received splenic T cells from 3–4 month old C57BL/6 wild-type mice. Thus, immune reconstitution of C57BL/6 mice with splenic T cells from young wild-type mice offered greater protection from progressive growth of 5T33L myeloma cells than did reconstitution with splenic T cells from old mice.

**Conclusions:**

Our findings support the hypothesis that age-associated changes in splenic T cell function contribute to the increased growth of 5T33 MMM cells in old compared to young C57BL/6 mice. Should similar processes occur in humans, increasing the anti-myeloma activity of T cells in old patients with multiple myeloma or transferring cryopreserved, young, autologous, T cells might benefit elderly patients with multiple myeloma.

## Background

The frequency of human cancer, in general, and in multiple myeloma, in particular, increases exponentially with age through the eighth decade of life [1,2]. A similar phenomenon has also been observed in experimental animals. Thus, pristane induces more plasmacytomas in old than in young mice [3]. Furthermore, the exponential increase in cancer with age implies that two or more age-associated factors contribute to the increased rate of development of neoplasms late in life. One age-associated factor in carcinogenesis is the accrual of genetic aberrations associated with the sequential transition of normal tissues to benign neoplasms and then to malignant neoplasms [4]. The link between a sequential appearance of genetic aberrations and neoplastic transformation has been extensively studied in human colon cancer where over decades the accrual of genetic aberrations in colonic epithelial cells leads to the transition from early to late adenomas and then to carcinomas [5]. A second factor, immune senescence, associated with impaired T cell function, has been reported to compromise immune surveillance and favor the growth and dissemination of neoplasms with increasing age [6]. Increasing experimental support suggests that tumors arise and progress more frequently in immunologically-compromised hosts although direct evidence that the age-associated decline in immune surveillance contributes to the exponential increase in tumor growth late in life has been difficult to demonstrate [7-9].

We have studied the growth of 5T33 MMM cells in C57BL/6 mice because myeloma in mice as in humans is a highly age-dependent cancer [10]. The 5T33 MMM cell line was derived from a spontaneous tumor that arose in an old, C57BL/KalwRij mouse and subsequently was adapted to grow in tissue culture [11]. We and others have studied the growth of 5T33 MMM cells in C57BL/6 mice because a number of mutant lines of this mouse strain are available from the Jackson Laboratories (Bar Harbor, Maine) and because old and young C57BL/6 mice are available from a colony maintained by the National Institute on Aging [12]. We now report that the age-associated increase in the growth of 5T33 MMM cells in old C57BL/6 mice reflects impaired immune surveillance associated with senescence of T lymphocytes.

## Results

### Growth of 5T33 MMM cells in C57BL/6 mice is influenced by age and T lymphocyte immune senescence

**Figure 1:**
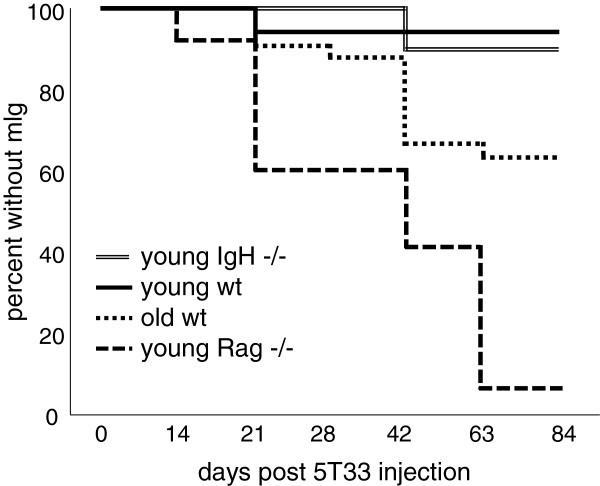
**Growth of 5T33-MMM cells was measured by the presence of 5T33-specific IgG2b mIg in serum. **Ten million HBSS-washed cultured 5T33 MMM cells were injected into 4 groups of mice on day 0: Group 1 included 18 young (2-3-month old), wild-type, C57BL/6 mice; Group 2 included 10 young (2–3 month-old) B-cell-deficient IgH^-/- ^(B6.129S2-Igh-6^tm1Cgn^/J) C57BL/6 mice; Group 3 included 34 aged (18–20 month old) wild-type C57BL/6 mice; and Group 4 included 28 young (2-3-month-old) Rag1-deficient C57BL/6 B-cell and T-cell deficient mice (B6.127S7-Rag1^tm1Mom^/J). The difference in percentages between mice with mIg in young RAG-1 deficient and old wild-type C57BL/6 mice and between old wild-type C57BL/6 and B-cell deficient or young wild-type C57BL/6 mice were statistically different (Kaplan-Meier statistic, p < 0.021).

The growth of 5T33 MMM cells in C57BL/6 female mice is reflected in the level of the specific monoclonal IgG2B produced by the 5T33 MMM cells that can be detected by serum protein electrophoresis (SPEP). We injected 5T33 MMM cells intravenously into four groups of female C57BL/6 mice: (1) wild type C57BL/6 3–4 month old; (2) wild type18–20 month old C57BL/6 mice; (3) 3–4 month old T- and B-cell deficient C57BL/6 mice, and (4) B-cell deficient (IgH-/IgH-) C57BL/6 mice. The rate and frequency of development of the mIgG2B serum monoclonal band, detected by SPEP, differed among the four groups (Figure 1).

The group of mice that developed the mIg band most rapidly and at the highest frequency was made up of young Rag1-deficient mice. This group also developed the highest percentage of mice with the mIg band (93% of 28 mice). The group that was next most rapid to develop the mIg band was the 18–20 month old wild type C57BL/6 mice. This group also had the second highest percentage of affected mice (35%). The groups least susceptible to developing the mIg band were young wild type C57BL/6 mice (6%) and the B-cell deficient young C57BL/6 mice (10%). The differences between the two fastest and two slowest groups to develop the mIg band were significant (Kaplan-Meier statistic p < 0.021). Together, these studies suggest that aging and the consequent senescence of T cells, or T cell deficiency at any age, increases susceptibility to the growth of the 5T33 myeloma.

Another potential factor influencing the appearance of the 5T33 mIg is the innate susceptibility of old mice to develop spontaneously mIgs. As we previously reported, approximately 50% of 18–20 month old wild-type C57BL/6 mice spontaneously developed age-associated serum mIg that differed in mobility and isotype from the mIg produced by 5T33 MMM cells [13,14]. It was not known whether 18–20 month old C57BL/6 mice with age-associated mIg were more susceptible to progressive 5T33 MMM cell growth following injection into old C57BL/6 mice and we found no significant difference in the frequency of 5T33 MMM cell-specific mIg in 18–20 month old C57BL/6 mice with (6/15) or without (6/19) spontaneous, age-associated mIg.

### IgH CDR3 analysis detects 5T33 cells in the spleen of sick mice without 5T33-specific mIg in their serum

A small number of Rag1-deficient and old wild type C57BL/6 mice appeared ill 2 to 3 months after injection of 5T33 cells but did not have detectable serum 5T33 mIg. It was possible that 5T33 MMM cells were present albeit not in sufficient numbers to produce a detectable serum 5T33-specific mIg although they caused morbidity in the mice. We used IgH CDR3 mRNA sequence and length analysis to identify 5T33 MMM cells within the spleen producing 5T33 MMM mRNA. This more sensitive technique we had used in detecting the spleen cells from 18 month old mice producing age-associated mIg.

Two of 28 young C57BL/6 Rag1-deficient mice injected with 5T33 MMM cells did not express a 5T33-specific serum mIg (Figure 1). These mice, despite showing weight loss and other signs of tumor morbidity, did not have a detectable IgG2b band on SPEP. However, when these animals were sacrificed, IgG2b CDR3 mRNA analysis revealed that both of these mice expressed the canonical CDR3 length and sequence of the rearranged 5T33 IgG2b genes in their spleens. Thus, 100% of Rag1-deficient mice supported the growth of 5T33 cells using this more sensitive technique although 2 of the 28 mice did not have detectable serum 5T33 mIg.

In a second experiment 8 of 17 18–20 month old wild type C57BL/6 mice did not develop 5T33-specific serum mIg. Two of these 8 mice expressed 5T33-specific IgG2b CDR3 RNA in the spleen. Thus, 11 of 17 (nearly 65%) of this group of 18–20 month old C57BL/6 mice supported the growth of 5T33 MMM cells when measured by IgG2b CDR3 mRNA analysis. Importantly, analysis showed that IgG2b CDR3 mRNA analysis detected a greater susceptibility of Rag-1 deficient 3–4 month old C57BL/6 mice (100% 5T33 MMM cells positive) compared to 18–20 month old C57BL/6 wild type mice (65% 5T33 MMM cells positive).

### IgH CDR3 analysis can be used to detect 5T33 cells in the spleen before serum mIgG is detectable

**Figure 2:**
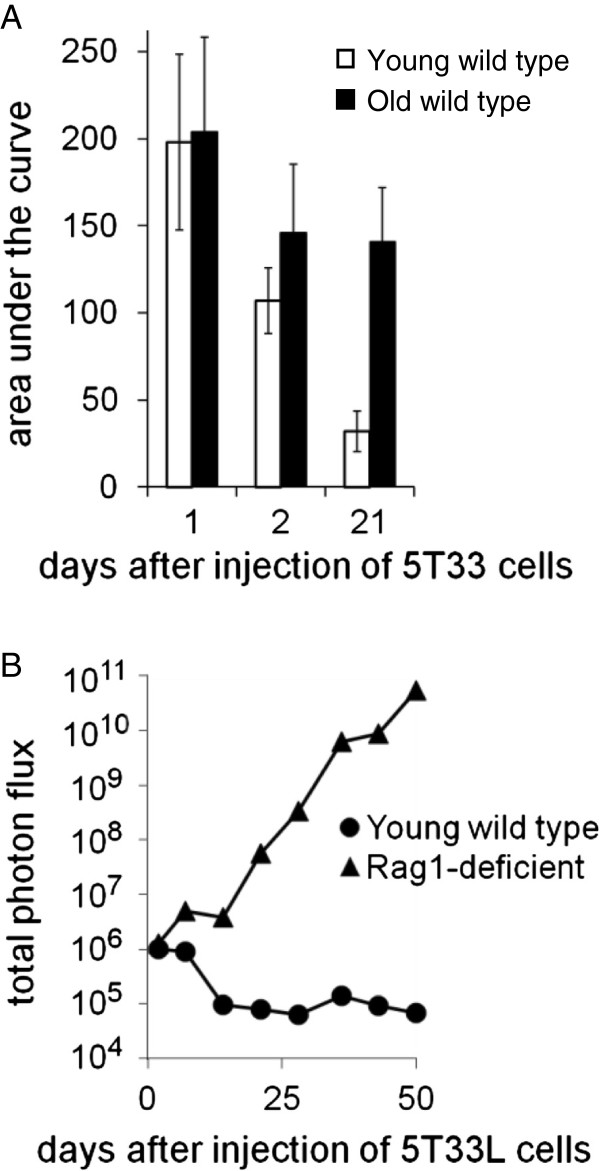
**A. The number of 5T33 MMM cells in the spleen was estimated by the area of the IgH CDR3 mRNA peak with the length and sequence analysis of cDNA of 5T33 MMM cells using ImageJ Version 1.37 (NIH, Bethesda, MD). **Splenic samples were obtained by partial splenectomy, 1, 2, or, at sacrifice, 21 days after injection after injection of 5T33 MMM cells. Splenic 5T33 cell number in young wild type C57BL/6 mice decreased by nearly 50% between day 1 and 2 and by 70% between day 2 and 21 following the injection of 5T33 MMM cells. In contrast, in old mice decreased only 30% and less than 5% between day 1 and 2 and less than 5% between day 2 and 21. After 21 days the index of 5T33 MMM cells remaining in the spleen was 4 fold greater in old than in young mice (p=0.036, Mann-Whitney Rank Sum Test). **B. **The average growth of 10 million luciferase-transfected 5T33 MMM (5T33L) cells injected into 3 young wild-type C57BL/6 mice or into 5 C57BL/6 Rag1-deficient mice by the average photon flux during the 7 weeks following the injection of 10 million 5T33L MMM cells. The average photon flux was measured using an IVIS optical imaging system after 20 minutes, 2 days, and at weekly intervals following 5T33L MMM cell infection for 7 weeks. Within 14 days the difference in the average photon flux of the C57BL/6-Rag-1-deficient and C57BL/6 wildtype mice was significantly different (p < 0.036 Wilcoxin-Mann-Whitney rank sum test) and increased progressively thereafter.

Fewer than 50% of Rag1-deficient mice injected with 10 million 5T33 MMM cells had detectable serum 5T33 mIg within 4 weeks of the injection of 5T33 MMM cells (Figure 1). However, when IgH CDR3 mRNA length and sequence analysis were used to identify 5T33 MMM cells in the spleens, most mice had detectable splenic 5T33 MMM cells before they had developed the 5T33-specific serum mIg. When young and old mice were injected with 10 million 5T33 myeloma cells and a partial splenectomy was performed one or two days later, all mice had 5T33 MMM cells in the spleen. However, the number of 5T33 MMM cells had significantly decreased within 21 days after injection into young but not in old mice based on 5T33 IgH CDR3 mRNA analysis (Figure 2A). The survival index of 5T33 MMM cells is represented by the area of an individual splenic 5T33-specific IgH CDR3 mRNA peak. In 10 young mice the average survival index progressively declined following 5T33 MMM cell injection. In contrast, in old mice the average survival index did not change significantly between the 2^nd^ and the 21^st^ day following the injection of 5T33 MMM cells. Consequently, 21 days following the injection of 5T33 MMM cells, the average 5T33 cell IgG2b survival index was significantly higher in old than in young C57BL/6 wild-type mice (Wilcoxin-Mann–Whitney rank sum test p < 0.036).

### Development of a 5T33 line constitutively expressing the firefly luciferase gene

As guidelines for animal experimentation limit the number and frequency of survival surgeries, it was not possible to monitor the mass of 5T33L MMM cells after injection by repeated partial splenectomies. To overcome this limitation, we created a line of 5T33 MMM cells stably transfected with the firefly luciferase gene that we termed 5T33L MMM cells that constitutively expressed the firefly luciferase gene. After the injection of 5T33L MMM cells, it was possible to repeatedly measure 5T33L MMM cell load in the same mice over long periods of time (Figure 2B). Two days after the injection of 10 million 5T33L MMM cells, photon flux following the injection of luciferin was the same in both young and Rag1-deficient C57BL/6 mice, confirming the findings using IgH CDR3 mRNA length analysis. However, by 5 days after injection of the 5T33L MMM cells and thereafter the load of 5T33L MMM cells began to diverge in young, wild type as compared to young, Rag1-deficient C57BL/6 when measured by total photon flux. Fourteen days after the injection of 5T33L into young, wild type C57BL/6 mice the photon flux has fallen to background levels comparable to photon flux from mice that had not received any 5T33L cells. There was no change in the background photon flux in young wildtype C57BL6 mice during the subsequent 6 weeks of observation. This is consistent with observations using 5T33-specific CDR3-mRNA peak area observed during the first 3 weeks following the injection of 5T33L MMM cells (Figure 2A). In contrast, the photon flux from 5T33L MMM cells increased exponentially between 14 and 50 days after 5T33L MMM cell injection into Rag1-deficient mice (Figure 2B). As early as day 14, the mean total flux of all five Rag1-deficient mice (4.0 × 10^6^ +/- 7.9 × 10^6^) was significantly greater (p = 0.036, Wilcoxon Mann–Whitney rank sum test) than that of the young wild type mice (9.5 × 10^4^ +/- 1.7 × 10^4^).

### Selection of old C57BL/6 mice that resist 5T33 cell growth

Resistance of mice to progressive growth of 5T33 cells is related to T cell function. Thus, Rag1-deficient mice that lack T and B cells were the most susceptible to 5T33 cell growth among mice that we have studied. Old mice, while less susceptible than Rag1-deficient mice, are more susceptible to growth of 5T33 cells than young or B-cell deficient mice (Figure 1). This suggests that immune senescence compromises T-cell function required for protection from 5T33 cell growth. Although T cell function declines with age, not all old animals are compromised to the same extent. This likely explains why more than a third of the old C57BL/6 old wildtype mice we studied resisted the growth of 5T33 cells. This was consistent with the hypothesis that about one third of old mice had better preserved T-cell immunity that explained their resistance to repeated challenge with 5T33 cells.

To investigate whether colonization of mice by 5T33 cells was a random event or reflected a characteristic of individual mice, we repeatedly challenged a group of 34 18–20 month old C57BL/6 mice with 10 million 5T33 cells. Fifteen of these 34 mice allowed the growth of 5T33 cells as determined by the presence of the 5T33-specific mIg in serum or, in its absence, the 5T33-specific CDR3 mRNA peak. Twelve of the mice that did not support the growth of 5T33 cells were rechallenged with the same dose of 5T33 cells. Only one of these 12 old mice supported 5T33 cell growth. The remaining 11 mice were challenged for a third time with ten million 5T33 cells and none of these mice supported the 5T33 cell growth. These observations suggested that resistance to 5T33 growth was a constitutional capacity of certain old mice and not a random event, such as the injection of different numbers of 5T33 MMM cells, extrinsic to individual old mice.

### Spleen cells from young, wild-type C57BL/6 mice inhibit the growth of 5T33L MMM cells in Rag1-deficient C57BL/6 mice

**Figure 3:**
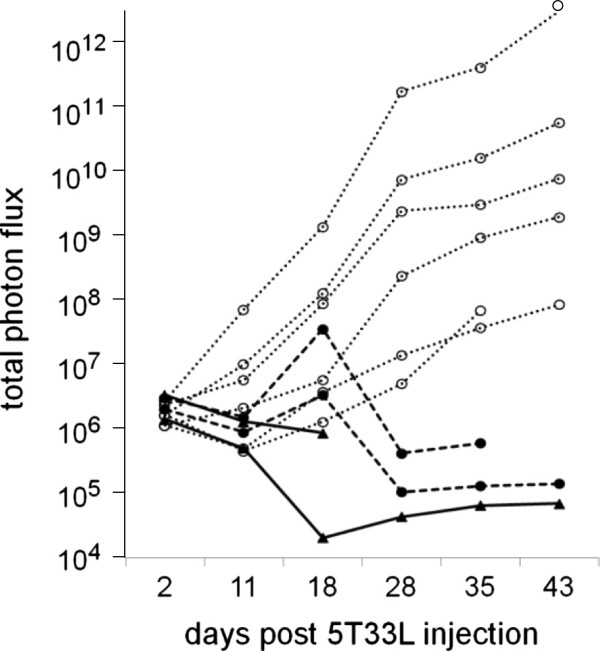
**Growth of 5T33L MMM cells were inhibited in two groups of 2 C57BL/6 Rag1-deficient mice given either 5 million (----) or 20 milllion (**___**) HBSS-washed spleen cells from 3 month-old C57BL/6 mice given 1 week before the injection of 10 million 5T33L MMM cells compared to two experiments in which a total of 6 C57BL/6 Rag1-deficient mice received only HBSS (**^**….**^**).** Bioluminescent imaging of photon flux (photons/sec) was measured 20 minutes of the injection of luciferin and after 2, 11, 18, 28, 35, and 43 days after the injection of 5T33L MMM cells. The difference between mice given and not given spleen cells was statistically significant and this difference increased progressively between 16 and 42 days after injection of the 5T33L cells (Chi-square-Fisher exact test p < 0.005).

As discussed on page 5, 5T33 MMM cells grew in all young, Rag1-deficient C57BL/6 mice. However, injection of 5 to 20 million spleen cells from young, wild-type C57BL/6 mice into young, Rag1-deficient C57BL/6 mice 1 week before the injection of 10 million 5T33 MMM cells inhibited the growth of 5T33L MMM cells (Figure 3). It should be noted, however, that in both mice given only 5 million spleen cells from young, wild-type C57BL/6 mice there was an early proliferation of 5T33L MMM cells before the growth of the 5T33L MMM cells was suppressed (compare heavy dashed lines with heavy solid lines in Figure 3). Similar results were observed in a second experiment.

In contrast, complete protection from 5T33L MMM cell growth was seen in all 4 young Rag1-deficient C57BL/6 mice injected with 20 million spleen cells from young wild-type C57BL/6 mice. Incomplete protection was seen in young Rag-1 deficient mice with only 5 of 14 young Rag1-deficient C57BL/6 mice given 5 million spleen cells from young, wild-type, C57BL/6 mice eliminating all 5T33L MMM cells. No protection from 5T33L MMM cell growth was seen in 3 young Rag1-deficient C57BL/6 mice reconstituted with 1 million or no spleen cells from young, wild type, C57BL/6 mice.

### Young splenic T cells protected Rag1-deficient C57BL/6 mice from 5T33L MMM cell growth better than old splenic T cells

**Figure 4:**
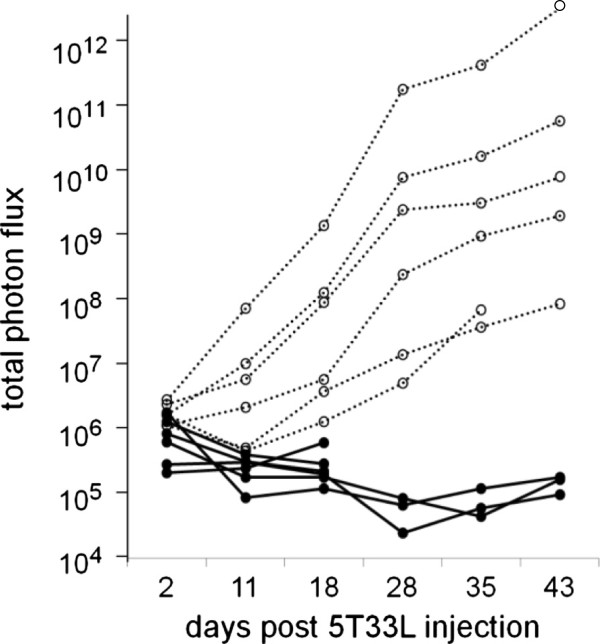
**Inhibition of the growth of 10 million 5T33L MMM cells injected into 6 C57BL/6 Rag1-deficient mice by 3 million HBSS-washed splenic T cells from 3 month old C57BL/6 mice 1 week earlier (**___**) or in two experiments including a total of 6 C57BL/6 Rag1-deficient mice given no purified splenic T cells from wild-type C57BL/6 mice had progressive tumor cell growth (**^**….**^**).** One week later all C57BL/6 Rag1-deficient mice were given 10 million 5T33L MMM cells. Photon flux was measured in all 12 mice 20 minutes after luciferin and 2, 11, 18, 28, 35, and 43 days after the injection of 5T33L MMM cells. Photon flux from C57BL/6 Rag1-deficient mice given splenic T cells was significantly less than photon flux from mice given only HBSS and this difference increased progressively between 18 and 43 days after injection of the 5T33L cells (Chi-square-Fisher exact test p < 0.0025).

The greater susceptibility of Rag1-deficient mice compared to B-lymphocyte-deficient C57BL/6 mice to the growth of 5T33L MMM cells suggested that impaired T cell function was the key to the greater susceptibility of Rag1-deficient mice to 5T33L MMM cells. To prove this hypothesis, purified splenic T cells (>95% CD3+) were prepared by negative selection from the spleens of 3–4 month old, wild-type C57BL/6 mice using the “Easy-Sep” T cell enrichment kit as described in the methods section. All 6 young Rag1-deficient C57BL/6 mice given 3 million purified splenic T cells from young wild-type C57BL/6 mice 1 week before the injection of 10 million 5T33L myeloma cells were protected from the growth of 5T33L MMM cells (Figure 4). In contrast, all 6 young Rag1-deficient C57BL/6 mice given no purified splenic T cells from wild-type C57BL/6 mice had progressive tumor cell growth.

Next, we determined the effect of age on the capacity of splenic T cells to protect Rag 1-deficient C57BL/6 mice from a challenge with 10 million 5T33L MMM cells. Three million, 1.5 million or 0.75 million splenic T cells from 3–4 month or from 18–20 month old wild-type C57BL/6 mice were injected into groups of 5 Rag-1-deficient young mice before being challenged 1 week later with 10 million 5T33L MMM cells. One group included 12 Rag1-deficient young C57BL/6 mice which received no T cells before being challenged with the injection of 10 million 5T33L MMM cells. The second group was divided into 3 groups of 5 young, Rag1-deficient C57BL/6 mice given either 0.75, 1.5 or 3.0 million splenic T cells from 18–20 month old C57BL/6 wild-type mice 1 week before the challenge with the an injection of 10 million 5T33L MMM cells. The third group was divided into 3 groups of 5 young, Rag1-deficient C57BL/6 mice given either 0.75, 1.5 or 3.0 million splenic T cells from 3–4 month old C57BL/6 wild-type mice 1 week before the challenge with the an injection of 10 million 5T33L MMM cells.

**Figure 5:**
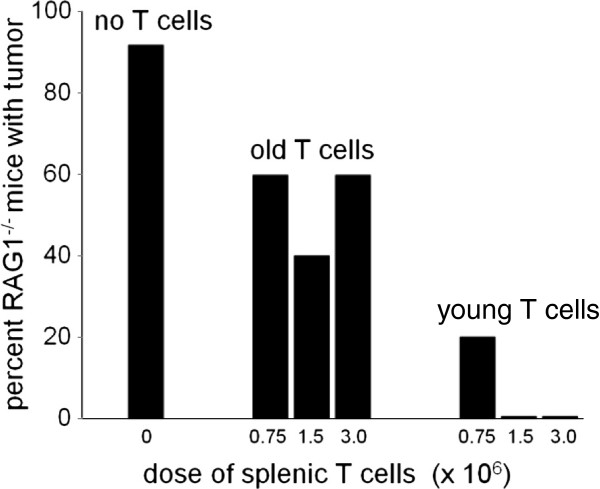
**Groups of 5 young Rag1-deficient C57BL/6 mice were given either 0.75, 1.5, or 3.0 million, wild-type splenic T cells from 3–4 or 18–20 month old wild type C57BL/6 mice 7 days prior to being challenged with 10 million 5T33L MMM cells. **A third group of 12 Rag1-deficient C57BL/6 mice were given no wild type splenic T cells prior to being challenged with 10 million 5T33 MMM cells. Survival of C57BL/6 Rag1-deficient mice given splenic T cells from 3–4 month old mice was significantly greater following challenge with 10 million 5T33L MMM cells than that of C57BL/6 Rag1-deficient mice given T cells from 18–20 month old C57BL/6 1 week prior to challenge with 10 million 5T33L MMM cells (Chi square statistic with Fisher exact test p < 0.017). A significantly greater number (11 of 12) of young Rag1-deficient mice not given any splenic T cells died following challenge with 5T33L MMM cells than young Rag1-deficient mice given splenic T cells from old mice prior to challenge with 5T33 MMM cells. (Chi-square-Fisher exact test, p < 0.019). The inhibitory potency of young splenic T cells compared to old splenic T cells was estimated to be 8 to1.

The results of this study are shown in Figure 5. In the first group which included 12 young Rag1-deficient C57BL/6 mice not given any splenic T cells prior to challenge with 10 million 5T33L MMM cells, 11 of 12 (92%) mice showed progressive growth of 5T33L MMM cells, indicated by increasing levels of photon flux, death, or were sacrificed because of severe morbidity within 6 weeks. In group II, 8 of 15 mice (53%) which received spleen T cells from 18–20 month old wild-type C57BL/6 mice showed progressive growth of 5T33L MMM cells, indicated by increasing levels of photon flux, death, or were sacrificed because of severe morbidity within 6 weeks.

Statistical analysis of this experiment showed that the administration of T cells from 18–20 month old mice protected 3–4 month old mice significantly better from tumor challenge than mice given no T cells (Chi-square Fisher exact p = 0.043). However, most important, T cells from 3–4 month old mice protected young Rag1-deficient C57BL/6 mice significantly better than recipients of T cells from 18–20 month old mice (Chi-square Fisher exact p = 0.014).

## Discussion

The increased susceptibility of old C57BL/6 wild-type mice to 5T33 MMM cell growth is associated with the decreased capacity of splenic T cells from old compared to young, wild-type, C57BL/6 mice to protect C57BL/6 mice from the progressive growth of 5T33 MMM cells. Future studies will be necessary to determine whether young spleen T cells that appear to be more effective in controlling the growth of 5T33L MMM cell-induced disease than old spleen T cells reflect the increased activity of regulatory T cells in the spleen of old mice that impair the capacity of young cytotoxic T cells to control the growth of 5T33 MMM in C57BL/6 wild-type mice. An age-associated increase in regulatory T cells that inhibit cytotoxic activity of T cells from old mice and humans has been reported [15,16].

## Conclusions

We conclude that the increased proliferation of 5T33 MMM cells in old mice is due to the age-associated loss of the T cell function that eliminates 5T33 MMM cells. The mechanism of the immune senescent-associated loss of murine T cell capacity to eliminate 5T33 MMM cells remains to be established. Two alternatives come to mind: (1) an age-associated loss of anti-tumor activity of T cells, and/or (2) an age-associated suppression of T cell anti-tumor activity by regulatory T cells. Whatever the mechanism, successful reduction of 5T33 MMM-induced mortality in old C57BL/6 mice would be predicted to follow replacement of T cells in old mice with T cells from young mice.

## Material and methods

### 5T33 MMM cell growth

Cultured 5T33 MMM cells were obtained from Dr. Babatunde Oyajobi (University of Texas. San Antonio, TX 78229) and cultured in Iscove’s DMEM (Cellgro, Herndon, VA) containing 25 mM L-glutamine, 25 mM HEPES to which was added 10% fetal bovine serum (FBS) and 1% Primocin (InvivoGen, San Diego, CA) in a 37°C, 5% CO_2,_ humidified incubator. The cells were maintained at a concentration below 500,000 cells/mL. Prior to injection, these cells were washed in sterile Hank’s Balanced Salt Solution (HBSS), counted, and re-suspended in HBSS at a concentration of 50–100 million cells/mL. A total of 10 million 5T33 MMM cells was injected intravenously (IV) into mice in 0.1 to 0.2 ml of HBSS.

### Mouse husbandry and purchase

As male C57BL/6 mice fought when housed together, female mice, which could be housed 5 to a cage, were used in this study and maintained in a barrier facility at the Weill Cornell Medical College Research Animal Resource Facility. The growth of 5T33 MMM cells in four groups of female mice was studied: young (3–4 month old) or old (18–20 month old) wild-type C57BL/6 mice obtained from the colony of aging mice maintained by the National Institute on Aging at Harlan Laboratories and two strains of young (3–4 month old) mutant C57BL/6 mice B-cell-deficient C57BL/6.129S2-Igh-6^tm1Cgn^/J or B- and T-cell deficient (Rag1-deficient) C57BL/6.127S7-Rag1^1m1Mom^/J obtained from the Jackson Laboratory (Bar Harbor, ME). All animal-use protocols were approved by the Weill-Cornell Medical College Institutional Animal Care and Use Committee.

### Experimental animal procedures

Mice were anesthetized by the intraperitoneal (IP) injection of 0.01 ml containing 10 mg of ketamineHCL and 1 mg xylazineHCL per ml water/gm body weight before retro-orbital bleeding, sacrifice by cervical dislocation, or partial splenectomy. Partial splenectomy was performed by exposing the spleen through an abdominal incision and ligation of the lower third of the spleen with surgical thread. The peritoneal cavity was then closed with surgical sutures and the skin secured with surgical staples.

### Preparation of spleen cells or splenic T cells for injection

Spleens from 3–4 or 18–20 month old wild-type C57BL/6 mice were removed under sterile conditions and single cell suspensions prepared by passing the splenic tissue in RPMI 1640 (Cellgro, Herndon, VA) through a metal screen. Erythrocytes in the spleen cell suspension were lysed by exposure to distilled water for 15 seconds followed by the addition of a 10% volume of 10X phosphate buffered saline (PBS). Splenocytes were then collected by centrifugation and washed in sterile HBSS. Splenic T cells were purified by negative selection using a combination of biotinylated monoclonal antibodies to cell surface antigens on mouse hematopoietic cells linked to magnetic nanoparticles using the “EasySep mouse” T cell enrichment kit according to the manufacturer’s instructions (StemCell Technology, Vancouver, CA). Total spleen cells and purified splenic T cells were washed in HBSS, counted, and injected through the tail vein into 3–4 month old Rag1-deficient C57BL/6 mice 1 week before they were challenged with 10 million 5T33L MMM cells injected into a tail vein.

### Serum protein electrophoresis

Serum monoclonal immunoglobulins were detected by serum protein electrophoresis (SPEP) gels prepared on Gelbond® films (Cambrex, Rockland, ME) using 1% Seakem HE agarose in barbital buffer (Sigma-Aldrich, St. Louis, MO) cast between 70°C glass plates and allowed to cool to room temperature. The gels were then placed at 4°C for one hour in a humidified chamber prior to loading. Three μl of 2:1 serum sample:barbital buffer were loaded per lane of a loading strip (SPIFE Urine/CSF Protein Template, Helena Laboratories, Beaumont, TX). The gels were run at 100 V for 25 minutes in a Beckman-Coulter PEP apparatus (Fullerton, CA), fixed in a 20% acetic acid and 30% methanol solution, and air dried. The gels were then stained with a Paragon Blue solution (Beckman-Coulter, Fullerton, CA) and de-stained with sequential washes in 5% acetic acid, 20% acetic acid and 30% methanol followed by drying at 70°C. SPEP band density was quantified using ImageJ Version 1.37 (NIH, Bethesda, MD). The background-subtracted density of the mIg was divided by the density of the standard serum mIg from a Rag1-deficient C57BL/6 mice injected seven weeks earlier with 5T33 MMM cells.

### Vector plasmid

A cytomegalovirus (CMV) promoter was constructed by amplifying this promoter from the vector pcDNA3.1+/- (Invitrogen, Camarillo, CA) by PCR using a forward primer containing an XhoI site near the 5^′^ end: 5^′^-TACTCGAGCAGATATACGCGTTGACATTC-3^′^, and a reverse primer with a HindIII site near its 5^′^ end: 5^′^-ACAAGCTTTCTAGTTAGCCAGAGAGCTCTG-3^′^. The PCR product was digested with Xhol and HindIII, purified using the Qiaquick PCR purification kit (Qiagen, Valencia, CA), and inserted into the polylinker of the luciferase-expression vector pGL4.20 (Promega, Madison, WI). The final construct was confirmed by restriction enzyme and partial sequence analysis.

### Stable transfection of the luciferase gene

Ten million 5T33 MMM cells were suspended in 0.5 mL of HBSS and transfected by electroporating 40 μg of PstI-linearized construct of the luciferase gene using a Gene Pulser Xcell Total System (Bio-Rad, Hercules, CA) set at 240 V and 500 μF capacitance. After incubation at 37°C for 24 hours, puromycin (7 microgm/ml) was added to the culture and the transfected (5T33L) MMM cells were passed twice weekly at which time luciferase activity was monitored in a Moonlight 2010 luminometer (Analytical Luminescence Laboratory, San Diego, CA). After two weeks of selection in puromycin medium, luciferase expression in the 5T33L cell line became stable and the cells were thence forth grown in the absence of puromycin. The 5T33L cells and the parent 5T33 cells showed no significant differences in vitro or in vivo with respect to cell growth, expression of CD138, or secretion of the 5T33-specific IgG2b mIg. In addition, the IgH CDR3 cDNA length and nucleotide sequences in 5T33 and 5T33L cells were identical. In vivo, there was no difference in the interval between the injection of 10 million 5T33 or 5T33L cells into Rag1-deficient mice and the appearance of mIg in serum or of severe morbidity.

### Bioluminescent imaging

The 5T33L cells were injected into shaved mice, anesthetized with isofluorine gas, injected IP with 300 μg D-luciferin (Xenogen, Alameda, CA) in 200μL PBS, and put in a light-tight chamber of an IVIS™ optical Imaging System (Xenogen, Alameda, CA). Grayscale photographic images and pseudocolor bioluminescent images were acquired. The total photon flux (photons/second) from each mouse was determined using Xenogen Living Image software and graphed versus time using KaleidaGraph (Synergy Software, Reading, PA). Background total flux was measured in mice injected with luciferin but not with 5T33L cells.

### Nucleic acid preparation

RNA or DNA was prepared from 5–10 million spleen or bone marrow cells from mice as well as from cultured or 5T33 MMM cells and extracted using RNeasy® or DNeasy®Kits (Qiagen, Valencia, CA) respectively. Up to 5 μg of extracted total RNA was reverse transcribed in 20 μL reaction using Oligo(dT) primer and Superscript II reverse transcriptase (Invitrogen, Carlsbad, CA) according to the manufacturer’s protocol.

### IgH CDR3 mRNA and DNA length analysis

PCR products derived from the DNA and cDNA were prepared as previously described [12]. PCR products derived from DNA were made using a sense primer, specific for the J558 VH family (5^′^-AAGGCCACACTG ACTGTAGAC-3^′^), and an anti-sense primer, specific for the JH2 family (5^′^-GACTGT GAGAGTGGTGCCTTG-3^′^). For the PCR products made from cDNA, the same J558 VH forward primer was used with a C-region anti-sense primer from exon I of the gamma chain C regions (5^′^-GGGAARTAVCCYTTGACMAGGCAYCC-3^′^). Reaction conditions were identical to those for the DNA reaction. Presence of PCR products was confirmed by electrophoresis on 1.5% agarose gels and visualization with ethidium bromide.

CDR3 length analysis was done as previously described (13). DNA or cDNA PCR products (2 ul) were used as substrates for run-off PCR reactions with a 5T33-specific fluorescent primer (5^′^-FAM CAAAGCAGTTACCATAAGCCCTCTC -3^′^). The elongation products were diluted with equal volume of loading buffer (95% formamide/10 mM EDTA), run on an acrylamide gel using an automated ABI 373A DNA sequencer and analyzed using Genescan 3.1 and Genotype 2.5 software (Applied Biosystems, Foster City, CA).

### Statistical analysis

Statistical analysis of differences among groups was analyzed using the Kaplan-Meier statistic, the Wilcoxon-Mann–Whitney rank sum test, or the Chi square statistic evaluated by the Fisher exact test.

## Competing interests

The authors declare that they have no competing interests.

## Authors’ contributions

AG performed the CDR3 analyses in the RAG-deficient mice. He also performed mIg analyses and participated in the cloning of the luciferase expression vector with YSS. YSS cloned the luciferase expression gene and created the 5T33L cell line by transfection. She also performed the assays of mice that received 5T33L cells. BH continued the mIg work and obtained the data about the mIg appearance after injection in the different mice. Brian also did CDR3 analysis of splenic mouse lymphocytes following 5T33 injection in young and old. JL cloned and sequenced the 5T33 CDR 3 region from which the primers for the CDR3 length assay were developed. He developed the mIg assays. PZ developed and supervised photon emission by mouses injected with 5T33L cells. CF carried out statistical analysis of data presented. RP contributed expertise on myeloma and design of the experimental model using cultured 5T33 myeloma to test the effect of T cell age on the resistance to 5T33 myeloma challenge. PS designed molecular studies and designed and produced the luciferase labeled 5T33 cells and directed all laboratory work. MEW designed the experimental strategy to measure the effect of age on T cell-mediated protection of immunodeficient mouse recipients of 5T33 murine myeloma cells. He led the writing of the manuscript. All authors read and approved the final manuscript.

## References

[B1] Mdzinarishvili T Sherman S Weibull-like Model of Cancer Development in Aging Cancer Inform 2010 9 179 188 20838610 10.4137/cin.s5460 PMC2935819

[B2] Waxman AJ Mink PJ Devesa SS Anderson WF Weiss BM Kristinsson SY McGlynn KA Landgren O Racial disparities in incidence and outcome in multiple myeloma: a population-based study Blood 2010 116 5501 5506 10.1182/blood-2010-07-298760 20823456 PMC3031400

[B3] Sato K Bloom ET Hirokawa K Makinodan T Increased susceptibility of old mice to plasmacytoma induction J Gerontol 1986 41 24 29 10.1093/geronj/41.1.24 3484487

[B4] Vogelstein B Kinzler KW Cancer genes and the pathways they control Nat Med 2004 10 789 799 10.1038/nm1087 15286780

[B5] Rajagopalan H Nowak MA Vogelstein B Lengauer C The significance of unstable chromosomes in colorectal cancer Nat Rev Cancer 2003 3 695 701 12951588 10.1038/nrc1165

[B6] Castle SC Uyemura K Fulop T Makinodan T Host resistance immune responses in advanced age Clin Geriatr Med 2007 23 463 479 10.1016/j.cger.2007.03.005 17631228 PMC7135540

[B7] Dunn GP Old LJ Schreiber RD The immunobiology of cancer immunosurveillance and immunoediting Immunity 2004 21 137 148 10.1016/j.immuni.2004.07.017 15308095

[B8] Sharma S Dominguez AL Lustgarten J High Accumulation of T regulatory cells prevents the activation of immune responses in aged animals J Immunol 2006 177 8348 8355 17142731 10.4049/jimmunol.177.12.8348

[B9] Swann JB Smyth MJ Immune surveillance of tumors J Clin Invest 2007 117 1137 1146 10.1172/JCI31405 17476343 PMC1857231

[B10] Radl J Croese JW Zurcher C Van den Enden Vieveen MH de Leeuw AM Animal model of human disease Am J Pathol 1988 132 693 697 PMC1880745 3414786

[B11] Manning LS Berger JD O’Donoghue HL Sheridan GN Claringbold PG Turner JH A model of multiple myeloma: culture of 5T33 murine myeloma cells and evaluation of tumorigenicity in the C57BL/KalwRij mouse Br J Cancer 1992 66 1088 1093 10.1038/bjc.1992.415 1457349 PMC1978028

[B12] Fowler JA Mundy GR Lwin ST Lynch CC Edwards CM A murine model of myeloma that allows genetic manipulation of the host microenvironment Dis Model Mech 2009 2 604 611 10.1242/dmm.003160 19779066 PMC2776114

[B13] LeMaoult J Delassus S Dyall R Nikolic-Zugic J Kourilsky P Weksler ME Clonal expansions of B lymphocytes in old mice J Immunol 1997 159 3866 3874 9378974

[B14] Szabo P Li F Mathew J Lillvis J Weksler ME Evolution of B-cell clonal expansions with age Cell Immunol 2004 231 158 167 10.1016/j.cellimm.2005.01.002 15919380

[B15] Lages CS Suffia I Velilla PA Huang B Warshaw G Hildeman DA Belkaid Y Chouguet C Functional Regulatory T Cells Accumulate in Aged Hosts and Promote Chronic Infectious Disease Reactivation J Immunol 2008 181 1835 1848 18641321 10.4049/jimmunol.181.3.1835 PMC2587319

[B16] Pan XD Mao YQ Zhu LJ Lie J Xie Y Wang L Zhang GB Changes of Regulatory T cells and Fox P3 Gene Expression in the Aging Process and its Relationship with Lung Tumors in Humans and Mice Chin Med J 2012 125 2004 2011 22884069

